# Living Environment Considerations on Obesity Prevention Behaviors and Self-Efficacy among Chinese Americans

**DOI:** 10.3390/ijerph18179322

**Published:** 2021-09-03

**Authors:** Doreen Liou, Jessica A. Karasik

**Affiliations:** Department of Nutrition and Food Studies, Montclair State University, Montclair, NJ 07043, USA; karasikj2@montclair.edu

**Keywords:** living environment, obesity risk reduction, Chinese Americans, self-efficacy

## Abstract

The aim of this study is to ascertain if the living environment (type of residential neighborhood and number of household members) will elucidate differences in obesity risk reduction behaviors and self-efficacy in Chinese Americans. A cross-sectional survey design was used to recruit participants from Los Angeles County and New York City metropolitan areas. A total of 650 adults were recruited from diverse socioeconomic backgrounds. Descriptive statistics were measured for 19 behaviors reflecting food intake and portion size control and items measuring self-efficacy and attitudes. *T*-tests were applied for the two categories of living environment. The mean age of the sample was 36.3 years. The ‘high income’ neighborhood group indicated a greater frequency of behaviors, including choosing steamed over fried foods (*p* < 0.01) and using small amounts of oil (*p* < 0.05). In general, this group exhibited more favorable attitudes and stronger self-efficacy to perform health behaviors. Multiple regression analyses point to the impact of self-efficacy in predicting behaviors. Nutrition professionals must assess client’s living environments in the adoption of obesity prevention behaviors and the fostering of behavioral confidence.

## 1. Introduction

The prevalence of obesity is expanding worldwide, requiring a multifaceted and culturally relevant approach across diverse ethnic populations with varying socioeconomic status [1]. Weight gain among adults is a health issue affecting Asian Americans in the United States [2]. Generational research points to rising obesity rates among successive generations of Chinese Americans [3,4,5,6,7]. In the past two decades, weight gain is more pronounced in U.S.-born Asians than their foreign-born counterparts [8]. Environmental influences and acculturation to American eating patterns exacerbate the risk of obesity for this population group. Asians born in the U.S. are 3–4 times more likely to be obese than individuals born overseas [9]. A positive correlation is detected between the length of U.S. residence and weight gain [10,11]. Asians tend to accumulate excess visceral fat, thereby increasing the risk for heart disease, chronic inflammation, and hypertension [12,13]. Asian Americans have a 30% to 50% increased likelihood to be diagnosed with type 2 diabetes than their white counterparts after adjusting for age and gender [9,14].

Asian Americans comprise approximately 5.4% of the U.S. population, with a significant predicted increase to 9.3% by 2060 [15]. California (Los Angeles county) and New York constitute the highest number of Asian Americans, including Chinese Americans, as this ethnic population is the largest Asian subgroup and one of the fastest-growing minority groups [16]. It is estimated that both these states comprise over 25% of Chinese Americans living in the country. Researchers have reported a higher frequency of adoption of obesity risk reduction behaviors among Chinese Americans living in Los Angeles county as compared with those living in the East Coast of the United States [17].

Researchers have addressed the impact of individual and neighborhood socioeconomic status on behaviors related to healthy living and obesity prevention [18]. Individuals with limited salaries living in low-income counties in the U.S. are at increased risk for coronary heart disease and obesity [19]. The urban built environment can pose a challenge for adhering to a healthy diet, which can include access to fresh produce and nutrient-dense foods [20,21].

Household types may be pivotal in affecting weight gain and chronic disease occurrence in individuals [22]. Researchers have reported low nutrient-dense diets among single-person Asian households in contrast with multi-person households with two or more members [23]. Single-person households may tend to face barriers in securing fruits and vegetables due to purchasing small quantities of food [24,25], resulting in unhealthy diets, which can be worsened with decreasing income. Studies on multi-person households point to family meals as promoting greater consumption of fresh produce, less fast food, and less sugar-sweetened beverages [26,27]. Leroux, Moore, and Dube reviewed obesity interventions targeting social relational factors, such as social support and social networks [28], and recommend addressing social-ecological levels when analyzing health interventions.

### Theoretical Framework

The adoption of health behaviors can be conceptualized by theoretical frameworks in behavioral theory and social psychology. According to Ajzen [29], in the formulation of the Theory of Planned Behavior (TPB), attitude constitutes an individual’s behavioral beliefs weighted by one’s evaluation of the outcome. Attitude towards the behavior is one of the constructs that are predictive of an individual’s behavioral intention. Another predictor of intention is perceived behavioral control, reflecting an individual’s perceived degree of control over performing a specific action. Self-efficacy reflects an individual’s belief or confidence in their ability to perform a particular action by overcoming barriers [30,31]. When considering self-efficacy to perform obesity prevention behaviors, we can examine an individual’s willingness and ability to consume plant-based foods while reducing high-fat and sugary options.

Considering the adverse health consequences of obesity, investigating obesity prevention behaviors in the context of individuals’ social and physical environments is warranted. The purpose of this study was to determine if the living environment (e.g., type of residential neighborhood, and the number of household members) is a differentiating factor to consider when ascertaining behaviors, attitudes, and self-efficacy among Chinese Americans residing in California and the New York (NY) metropolitan area. In this investigation, the researchers chose to examine attitudes and self-efficacy and not the entire TPB framework, as a prior study documented their impact on obesity prevention behaviors [17].

The null hypotheses are that (a) typical frequency of the behavior is equal in the ‘high income’ and ‘middle-low income’ neighborhood groups, and (b) typical frequency of the behavior is equal in the single-person and multi-person households. The investigators hypothesize that (a) individuals living in ‘high income’ neighborhoods would report more frequent engagement of obesity prevention behaviors than their ‘middle-low income’ counterparts, and (b) respondents living in multi-person households would report more frequent engagement of behaviors than single-person households.

## 2. Materials and Methods

A cross-sectional survey design was used, which consisted of a convenience sample of Chinese Americans living in Los Angeles (LA) County, California and New York City, New York. Within these two states, the researchers strategically selected cities with the highest percentages of Chinese American residents. The U.S.- and foreign-born participants ranged between the ages of 18 to 60 years. Participants were solicited from Chinese American associations, cultural, educational, and religious institutions exhibiting a wide range of socioeconomic status and types of residential neighborhoods. The recruitment of at least 200 participants per sample ensured statistical power based on the number of variables measured. As an incentive, respondents were given an opportunity for a raffle drawing of USD 25 and USD 50 gift cards. Data was collected in LA County from January 2017 to June 2017 with a final sample size of 203. Prior data collection from the New York sample was completed between September 2012 to April 2013 with 447 Chinese Americans [32,33]. All qualified individuals were allotted a survey instrument, informed consent form, and a self-addressed, stamped envelope. The study was approved by the Institutional Review Board of Montclair State University (IRB# IRB-FY15-16-249; 24 April 2017). Findings from the LA sample were systematically compared with published data from a NY metropolitan study with 447 Chinese [32,33].

### 2.1. Questionnaire

The survey instrument contained 47 questions measuring obesity risk reduction behaviors, attitudes, self-efficacy, and demographic factors (Table 1). On average, the survey took 15 min to complete. A total of 19 behavioral questions measured five domains of health actions over the past month, anchored by “1” never/rarely and “4” always/usually. The domains included “eating context” (4 items), “food context” (9 items), “psychological context” (2 items), “physical activity” (2 items), and “knowledge awareness” (2 items). These domains were derived from a comprehensive literature review, along with the extraction of qualitative data and findings on Chinese Americans [34,35].

Based on the TPB, 12 items measured attitude towards behaviors using a 7-point Likert-type scale. For example, “Eating high-calorie junk foods is …” and “Eating home-cooked meals instead of restaurant-prepared foods is …” (‘favorable’ to ‘unfavorable’). Participants rated their self-efficacy (9 items) for the engagement of behaviors, such as selecting foods that are not fried and consuming small portion sizes of food. Response options to the self-efficacy items were indicated on 5-point scales (‘extremely confident’ to ‘not at all confident’).

Demographic factors were assessed, such as country of origin, age, gender, educational level, and marital status. Participants identified the number of individuals living in their household. Responses were grouped into single-person household (‘1’) versus multi-person household (‘2 or more individuals’). Respondents also described the neighborhood in which they lived (‘high income’ versus ‘middle-low income’).

### 2.2. Questionnaire Validity and Reliability

Face validity was ascertained via a pilot sample of 30 Chinese Americans who provided confirmation on the meaning and clarity of the questionnaire items. A research panel of nutritionists and collegiate professors reviewed the survey instrument for an accurate representation of theoretical constructs. Construct validity was established by an exploratory factor analysis of principal variables. The entire scale produced 9 distinct factors accounting for 62.3% of the variance in responses. After additional factor analysis for each subscale, 6 items had a factor loading of less than 0.40 and were deleted from the scale [36].

The subscale of obesity risk reduction behavior yielded 5 distinct factors accounting for 60.3% of the variance in responses. These distinct factors corresponded conceptually to the 5 contexts of obesity risk reduction behaviors: food, eating behavior, physical activity, psychological context, and knowledge/awareness. Reliability was measured using Cronbach’s alpha internal consistency assessment. The Cronbach’s alpha coefficients of the behavioral variables (0.8) and all the psychosocial variables were at or above 0.70, reflecting good psychometric properties. Further details of the instrument’s validity and reliability can be found in a previously published study [32].

### 2.3. Statistics

Behavioral, psychosocial, and demographic data were described using frequency distributions. Statistical Package for Social Science (SPSS), version 23.0, was the computer software used for data analysis. Independent sample *t*-tests were conducted to detect mean differences between the two neighborhood groups for the 19 behavioral items. In addition, *t*-tests were conducted among single versus multiple-member households. Stepwise multiple regression analyses provided an assessment of the variance explained for each of the 5 behavioral contexts. In each of the regression analyses, the independent variables included type of residential neighborhood, household type, income, self-efficacy, and attitude. In conducting statistical computations, a 0.05 significance level was established. Ten incomplete surveys were discarded by the researchers and were not used in the data analysis.

## 3. Results

### 3.1. Demographic Data

Approximately 1033 surveys (350 LA + 683 NY) were distributed to eligible participants in the Los Angeles County and the New York metropolitan area. A total of 650 questionnaires were returned (203 LA + 447 NY), accounting for a 63% response rate. The mean age of the LA study participants was 38.1 (SD = 12.8) and 35.6 (SD = 15.1) years old for the NY sample. As presented in Table 2, the mean age of the entire sample was 36.3 years, with 67% females.

A total of 285 respondents (43.8%) lived in ‘high income’ neighborhoods, and 365 individuals (56.2%) resided in ‘middle-low income’ areas. Approximately 50% of the entire sample had an annual household income of less than USD 40,000. As for educational attainment, 34.8% of the participants received a college degree. A total of 76 respondents (11.7%) lived in single-person households and 574 individuals (88.3%) reported living in multi-person households. Forty-eight percent of the respondents were married, and 47% were single individuals.

### 3.2. T-Test Comparison of Dietary Behaviors and Psychosocial Factors

#### 3.2.1. Type of Household

In examining single versus multiple-member households, multiple-member households reported a higher frequency of home-cooked meals instead of restaurant-prepared foods (*p* < 0.01) than their single counterparts.

#### 3.2.2. Type of Residential Neighborhood with Behavior

Individuals living in ‘high income’ neighborhoods reported a higher frequency of adoption of behavior, such as choosing steamed foods over fried ones (*p* < 0.01), using small amounts of cooking oils or fat (*p* < 0.05), eating at least three servings of vegetables per day (*p* < 0.05), eating at least two servings of fruit per day (*p* < 0.05), and eating healthy snacks (*p* < 0.05) (Table 3).

#### 3.2.3. Type of Residential Neighborhood with Attitude

In comparing the mean values of individuals living in ‘high’ versus ‘middle-low income’ neighborhoods, significant differences were seen in attitudes toward behavior. Respondents living in high income neighborhoods reported more favorable attitudes toward eating home-cooked meals (*p* < 0.001), choosing small portions of foods (*p* < 0.001), using small amounts of cooking oils or fat (*p* < 0.001), restricting intake of high-calorie drinks (*p* < 0.01), choosing steamed foods over fried ones (*p* < 0.001), and eating a lot of fruits and vegetables (*p* < 0.001).

#### 3.2.4. Type of Residential Neighborhood with Self-Efficacy

Significant differences in self-efficacy were detected in comparing the mean values of individuals living in ‘high’ versus ‘middle-low income’ neighborhoods. Higher levels of self-confidence were reported in respondents living in ‘high’ income areas, particularly for consuming small portion sizes of foods (*p* < 0.001), selecting foods that are not fried (*p* < 0.01), eating a lot of fruits and vegetables (*p* < 0.05), limiting high-calorie beverages (*p* < 0.05), making healthful choices at fast-food restaurants (*p* < 0.01), and eating healthy snacks (*p* < 0.05).

### 3.3. Multiple Regression Analyses

In predicting behavior (*eating context*), self-efficacy, attitude, and household type accounted for 25.8% of the variance explained for the outcome variable (Table 4). As for the variance accounted for *food context*, self-efficacy, attitude, and income contributed 35.9% in the regression model. Self-efficacy was the sole predictor of behavior (*psychological context*), accounting for 7.9% of the variability. As for predicting *physical activity context*, self-efficacy and attitude were influencing factors, amounting to 19.6% of the variance. Self-efficacy and attitude also accounted for 11.8% of the variance in the *knowledge awareness context* of behavior.

## 4. Discussion

This study reflects a multi-state approach investigating dietary behaviors conducive to obesity among Chinese Americans. It highlights a critical comparative analysis of individuals’ living environment (type of residential neighborhood and household) and psychosocial factors, such as attitudes toward behavior and self-efficacy. Even with the understood and noted link between living environment and health, there is limited research regarding this association among Chinese Americans.

In general, the sample of Chinese Americans living in ‘high income’ neighborhoods reported a higher frequency of behaviors related to obesity prevention when compared with those living in ‘middle-low income’ areas. Our research hypothesis was confirmed in the behaviors primarily reflecting *food context*, such as limiting the intake of high-calorie beverages and portion sizes of foods, consuming the recommended daily servings of fruits and whole grains, choosing steamed over fried foods, and selecting healthy snacks. One behavior within the *eating context*, namely, following traditional Chinese food patterns, also pointed to a greater adoption with the ‘high income’ neighborhood group.

Individuals living in ‘high income’ areas also reported more favorable attitudes toward eating steamed foods and home-cooked meals, selecting small portion sizes and amounts of cooking oils, and consuming a lot of fruits and vegetables. The attitude was a salient predictor in four out of five behavioral contexts used in multiple regression models.

The strong predictive power of self-efficacy has been evident in multiple regression models. Self-efficacy consistently accounted for the most variance, surpassing attitude, type of neighborhood, income, and type of household. Our sample of Chinese Americans living in ‘high income’ neighborhoods also reported greater confidence in performing behaviors. Prior studies have documented the solid predictive power of self-efficacy in the adoption and maintenance of dietary behavior and physical activity [37,38].

We surmise that affluent neighborhoods in the New York metropolitan area and Los Angeles county have accessible resources and facilities for securing fresh produce and other nutrient-dense food options. Leal and Chaix [18] indicated environmental characteristics associated with obesity, including low area socioeconomic position and low accessibility to supermarkets. They discussed the limited success of educational programs targeting attitudes stem from the failure to consider environmental barriers to healthy living. These barriers include low accessibility to supermarkets and low social cohesion.

Our study indicated that multi-person households reported a higher frequency of home-cooked meals than their single-person household counterparts. It is postulated that the presence of a spouse or children would enable home-cooked meals to be more economical and practical than eating out at restaurants. Other researchers point to the importance of targeting social relational factors, such as social support and social networks [22]. Many studies can attest to the impact that social and neighborhood environments have on affecting both obesity prevention and its intervention [39].

There are noteworthy strengths within our research endeavor. The recruitment of respondents from the East and West coasts of the U.S. reflected the diversity of socioeconomic and educational backgrounds. A multi-state comparative approach confirmed the importance of physical environments in the adoption of healthy dietary behaviors to mitigate obesity risk among an underrepresented population group of Chinese Americans in North America. This study fills an important gap in the literature by revealing contexts of health behaviors and attitudinal predispositions of Chinese individuals living in different residential neighborhoods. This study can open new avenues of inquiry among health professionals and policymakers and lead to impactful strategies for interventions.

The study limitations include the use of a non-randomized, convenience sample, which limits the ability to generalize the findings to the entire Chinese American population at large. Individuals who volunteered in this study may be more health-conscious than non-participants, this impacted self-reported attitudes and health behaviors. The collection of survey data between the two states of New York and California was implemented at different points in time, which may affect the reporting of beliefs and behavior stemming from possible geographic or economic influences. Despite these caveats, this study highlights potential opportunities for clinicians to intervene and explore the intricacies of behavioral practices conducive to obesity prevention.

Future studies can elucidate the environmental impact of neighborhoods, albeit the accessibility of fresh produce or the competitive availability of junk foods in specific regions and cities in the U.S.A. A longitudinal study involving repeated observations of the same variables can determine the stability of behaviors, attitudes, and self-efficacy in preventing obesity, especially in this ethnic group of Chinese Americans.

## 5. Conclusions

Health professionals and nutrition educators working with Chinese American clients living in ‘middle-low income’ neighborhoods need to address their confidence and ability to practice portion size control and select plant-based, whole foods in place of processed items high in fat and sugar. In fostering favorable attitudes or dispositions toward these dietary behaviors, creating a sense of empowerment and confidence to enact these behaviors can be reinforced by supportive physical and social environments.

## Figures and Tables

**Table 1:** Sample questionnaire items and constructs for obesity risk reduction behaviors.

Constructs	Sample Questionnaire Statements
Psychological context	In the past month, how often did you engage in the following behaviors: Took time to relax and improve my emotional well-being? (e.g., social involvement, positive thinking) Took time to relax to decrease the amount of stress I feel?
Physical activity context	Exercised at least 30 min, on 3 to 5 days per week (e.g., walking, biking)? Engaged in at least 1 physically active leisure activity?
Eating context	Ate home-cooked meals over restaurant-prepared foods? Ate smaller portion sizes of foods than usual? Followed traditional healthful Chinese food patterns (e.g., eating more fruits and vegetables, less red meat)? Used portion size control methods to help decide how much to eat?
Food context	Ate steamed foods instead of fried foods? Used some amounts of oils or fat when preparing or cooking foods? Ate at least 3 servings of vegetables per day? (1 serving = ½ cup cooked, 1 cup fresh leafy vegetables) Ate at least 2 servings of fruits each day? (1 serving = 1 medium fruit) Ate at least 3, 1-ounce servings of whole grains per day? Made healthier choices at fast food restaurants? Ate healthful snacks (e.g., fruit, nuts, etc.)? Ate healthful pre-packaged foods Limited intake of high calorie beverages (e.g., soft drinks, juice, alcoholic drinks)?
Knowledge awareness context	Monitored my body weight? Learned about obesity risk and prevention (e.g., attending seminars, reading health articles, watching health programs on TV)?
Psychosocial Statements—Attitudes	Eating home-cooked meals instead of restaurant-prepared foods is … (Favorable to Unfavorable)
Psychosocial Statements—Self-Efficacy	How confident are you in consuming small portion sizes of foods?

Adapted from Liou and Bauer [17].

**Table 2:** Socioeconomic data of LA sample and NY metropolitan sample.

Category	Total Sample (*n* = 650)
Gender %	
Male	33.0
Female	67.0
Age, years	
Mean	36.33
Range	18 to 60
Standard Deviation (SD)	14.46
Income, USD, %	
Under 20,000	36.4
20,000–39,999	14.1
40,000–59,999	16.7
60,000–79,999	11.0
80,000 and above	21.8
Neighborhood residence %	
High income neighborhood	43.8
Middle-low income neighborhood	56.2
Education %	
Elementary school or less	0.6
Some high school	2.2
High school graduate	9.2
Some college	18.3
College graduate	34.8
Post-graduate	32.6
Missing	2.3
Type of household %	
Single-person household	11.7
Multi-person household	88.3
Marital status %	
Married	48.0
Divorced	2.8
Separated	1.0
Domestic partner	1.0
Never married	47.2

**Table 3:** Comparisons on obesity prevention behaviors between ‘high’ and ‘middle-low income’ neighborhoods.

Category for *T*-Test	High Income Mean (*n* = 285)	SD	Middle-Low Income Mean (*n* = 365)	SD	Sig. (2-Tailed)
Psychological					
Took time to decrease the amount of stress I feel	2.71	0.89	2.57	0.91	0.058
Took time to relax and improve my emotional well-being	2.85	0.92	2.72	0.91	0.076
Physical Activity Context					
Engaged in at least 1 physically active leisure activity	2.62	1.09	2.48	1.09	0.092
Exercised at least 30 min, on 3–5 days/week	2.57	1.12	2.42	1.10	0.094
Eating Context					
Ate home-cooked meals instead of restaurant-prepared meals	3.11	0.84	3.05	0.91	0.39
Limited my portion sizes of foods	2.51	0.97	2.40	0.94	0.15
Used portion size control methods to help decide how much to eat	2.19	1.04	2.03	0.99	0.06
Followed traditional healthful Chinese food patterns	2.93	0.97	2.73	1.00	0.009 **
Food Context					
Ate steamed foods instead of fried foods	2.79	0.91	2.58	0.92	0.003 **
Used small amounts of oils or fat when preparing or cooking foods	3.08	0.93	2.88	0.99	0.010 *
Ate at least 3 servings of vegetables per day	2.84	0.91	2.66	0.92	0.012 *
Ate at least 2 servings of fruit each day	2.72	0.95	2.55	0.98	0.020 *
Ate at least 3 1-oz servings of whole grains per day	2.67	0.98	2.58	0.97	0.247
Made healthier choices at fast food restaurants	2.48	1.07	2.38	1.05	0.246
Ate healthful snacks	2.82	0.95	2.66	0.93	0.027 *
Ate healthful pre-packaged foods	2.32	1.02	2.19	0.99	0.126
Limited intake of high-calorie beverages	3.07	1.06	2.97	1.06	0.203
Knowledge Awareness Context					
Monitored my weight	2.65	1.07	2.49	1.04	0.059
Learned about obesity risk and prevention	2.02	1.05	1.99	1.05	0.732

* *p* < 0.05 level, ** *p* < 0.01 level.

**Table 4:** Psychosocial variables regressed on obesity risk reduction behaviors.

The Predicted	Significant Predictors	*R*^2^ (%)	*df*	*β*	*B*	SE of *B*	*p*-Value
Food context	1. Self-efficacy	35.9	3	0.57	0.50	0.03	<0.001
	2. Attitude		3	0.18	0.18	0.02	<0.001
	3. Income		3	−0.07	−0.03	0.01	0.023
Eating context	1. Self-efficacy	25.8	3	0.49	0.50	0.03	<0.001
	2. Attitude		3	0.14	0.10	0.03	<0.001
	3. Household type		3	0.06	0.04	0.02	0.047
Psychological context	1. Self-efficacy	7.9	1	0.28	0.36	0.05	<0.001
Physical activity context	1. Self-efficacy	19.6	2	0.44	0.64	0.05	<0.001
	2. Attitude		2	0.07	0.07	0.04	0.045
Knowledge awareness context	1. Self-efficacy	11.8	2	0.34	0.44	0.05	<0.001
	2. Attitude		2	0.08	0.07	0.04	0.035

## Data Availability

The data that support the findings of this study are available from the corresponding author, upon reasonable request.

## References

[1] World Health Organization Obesity. https://www.who.int/news-room/facts-in-pictures/detail/6-facts-on-obesity.

[2] Office of Disease Prevention and Health Promotion Nutrition, Physical Activity, and Obesity: Latest Data. https://www.healthypeople.gov/2020/leading-health-indicators/2020-lhi-topics/nutrition-physical-activity-and-obesity/data#NWS-9.

[3] Lv N., Brown J.L. (2010). Chinese American family food systems: Impact of Western Influences. J. Nutr. Educ. Behav..

[4] Kirshner L., Yi S.S., Wylie-Rosett J., Matthan N.R., Beasley J.M. (2019). Acculturation and diet among Chinese American immigrants in New York City. Curr. Devel. Nutr..

[5] Lutsey P.L., Roux A.V.D., Jacobs D.R., Burke G.L., Harman J., Shea S., Folsom A.R. (2008). Association of acculturation and socioeconomic status with subclinical cardiovascular disease in the Multi-Ethnic Study of Atherosclerosis. Am. J. Public Health.

[6] Demory-Luce D., Morales M., Nicklas T. (2005). Acculturation, weight status, and eating habits among Chinese-American preschool children and their primary caregivers: A pilot study. Nutr. Res..

[7] Argueza B.R., Sokal-Gutierrez K., Madsen K.A. (2020). Obesity and obesogenic behaviors in Asian American children with immigrant and US-born mothers. Int. J. Environ. Res. Public Health.

[8] Sing G.K., Ling S.C. (2013). Dramatic increases in obesity and overweight prevalence among Asian subgroups in the United States, 1992–2011. ISRN Prev. Med..

[9] Wang S., Quan J., Kanaya A.M., Fernandez A. (2011). Asian Americans and obesity in California: A protective effect of biculturalism. J. Immigr. Minor. Health.

[10] Lindberg N.M., Stevens V.J. (2013). Immigration and weight gain: Mexican-American women’s perspectives. J. Immigr. Minor. Health.

[11] Huang C.-C. (2019). The influence of acculturation and weight-related behaviors on body mass index among Asian American ethnic subgroups. Immigr. Health.

[12] Wong R.J., Ahmed A. (2014). Obesity and non-alcoholic fatty liver disease: Disparate associations among Asian populations. World J. Hepatol..

[13] Wei H., Zhang S., Song A., Yang M., Jiao J., Allison D.B., Heymsfield S.B., Zhu S. (2013). Greater abdominal fat accumulation is associated with higher metabolic risk in Chinese than in white people: An ethnicity study. PLoS ONE.

[14] Hsu W.C., Araneta M.R.G., Kanaya A.M., Chiang J.L., Fujimoto W. (2015). BMI cut points to identify at-risk Asian Americans for type 2 diabetes screening. Diabetes Care.

[15] United States Census Bureau Projections of the Size and Composition of the U.S. Population: 2014 to 2060. S. L. Colby & J. M. Ortman. (Current Population Reports). https://www.cencus.gov/library/publications/2015/demo/p25-1143.html.

[16] Asian Americans Advancing Justice—Los Angeles. A Community of Contrasts: Asian Americans, Native Hawaiians and Pacific Islanders in Los Angeles County. Asian Americans Advancing Justice—Los Angeles: Los Angeles County, CA, USA. https://advancingjustice-aajc.org/sites/default/files/2016-09/AAAJ_Western_Dem_2015.pdf.

[17] Liou D., Bauer K.D. (2018). Contrasting obesity-related beliefs and behaviors among West and East Coast Chinese Americans. J. Food Nutr. Res..

[18] Leal C., Chaix B. (2010). The influence of geographic life environments on cardiometabolic risk factors: A systematic review, a methodological assessment and a research agenda. Obes. Rev..

[19] Shaw K.M., Theis K.A., Self-Brown S., Roblin D.W., Barker L. (2016). Chronic disease disparities by county economic status and metropolitan classification. Behavioral Risk Factor Surveillance System, 2013. Prev. Chronic. Dis..

[20] Pinard C.A., Byker Shanks C., Harden S.M., Yaroch A.L. (2016). An integrative literature review of small food store research across urban and rural communities in the U.S. Prev. Med. Rep..

[21] Dean W.R., Sharkey J.R. (2011). Rural and urban differences in the associations between characteristics of the community food environment and fruit and vegetable intake. J. Nutr. Educ. Behav..

[22] Kim S., Hwang D.S. (2017). The study on the effects of lifestyle of single-person households and multi-person households on life satisfaction—According to preparation for old age. J. Consum. Cult..

[23] Lee J.Y., Choi S.K., Seo J.S. (2019). Evaluation on the nutrition status and metabolic syndrome prevalence of the members according to the number of household members based on the Korea National Health and Nutrition Examination Survey (2013–2014). Korean J. Community Nutr..

[24] Jeon Y., Ahn B. (2016). Food consumption behaviors according to household types. J. Rural Dev..

[25] Lee K.W., Shin D. (2021). Relationships of dietary factors with obesity, hypertension, and diabetes by regional type among single-person households in Korea. Nutrients.

[26] Newman S.L., Tumin R., Andridge R., Anderson S.E. (2015). Family meal frequency and association with household food availability in United States multi-person households: National Health and Nutrition Examination Survey 2007–2010. PLoS ONE.

[27] Schoeppe S., Vandelanotte C., Rebar A.L., Hayman M., Duncan M.J., Alley S.J. (2018). Do singles or couples live healthier lifestyles? Trends in Queensland between 2005–2014. PLoS ONE.

[28] Leroux J., Moore S., Dubé L. Beyond the “I” in the Obesity Epidemic: A review of Social Relational and Network Interventions on Obesity. https://www.ncbi.nlm.nih.gov/pmc/articles/PMC3770066/.

[29] Ajzen I. (1991). The theory of planned behavior. Organ. Behav. Hum. Decis. Process..

[30] Bandura A. (1986). Foundations of Thought and Action: A Social Cognitive Theory.

[31] Ajzen I. (2002). Perceived behavioral control, self-efficacy, locus of control, and the theory of planned behavior. J. Appl. Soc. Psych..

[32] Liou D., Bauer K.D., Bai Y. (2011). Psychosocial variables and obesity risk reduction behaviors in Chinese Americans. Ecol. Food Nutr..

[33] Liou D., Bauer K.D., Bai Y. (2016). A cross-sectional analysis of obesity risk reduction behaviors and demographic factors among Chinese Americans. J. Obes. Weight-Loss Medicat..

[34] Bes-Rastrollo M., Basterra-Gortari F., Sanchez-Villegas A., Marti A., Martinez J., Martinez-Gonzalez M. (2010). A prospective study of eating away-from-home meals and weight gain in a Mediterranean population: The SUN (Seguimiento Universidad de Navarra) Cohort. Public Health Nutr..

[35] Smith K.J., Blizzard L., McNaughton S.A., Gall S.L., Dwyer T., Venn A.J. (2011). Takeaway food consumption and cardio-metabolic risk factors in young adults. Eur. J. Clin. Nutr..

[36] Kline P. (2014). An Easy Guide to Factor Analysis.

[37] Schwarzer R., Schuz B., Ziegelmann J.P., Lippke S., Luszczynska A., Scholz U. (2007). Adoption and maintenance of four health behaviors: Theory-guided longitudinal studies on dental flossing, seat belt use, dietary behavior, and physical activity. Soc. Behav. Med..

[38] Liou D., Contento I.R. (2001). Usefulness of psychosocial theory variables in explaining fat-related dietary behavior in Chinese Americans: Association with degree of acculturation. J. Nutr. Educ..

[39] Suglia S.F., Shelton R.C., Hsiao A., Wang Y.C., Rundle A., Link B.G. (2016). Why the neighborhood social environment is critical in obesity prevention. J. Urban Health.

